# Photodynamic Physiology—Photonanomanipulations in Cellular Physiology with Protein Photosensitizers

**DOI:** 10.3389/fphys.2017.00191

**Published:** 2017-04-04

**Authors:** Hong Ning Jiang, Yuan Li, Zong Jie Cui

**Affiliations:** College of Life Science, Institute of Cell Biology, Beijing Normal UniversityBeijing, China

**Keywords:** photosensitization, protein photosensitizer, photonanomanipulation, singlet oxygen, calcium oscillation, pancreatic acinar cells, photopharmacology, ligand-independent

## Abstract

Singlet oxygen generated in a type II photodynamic action, due to its limited lifetime (1 μs) and reactive distance (<10 nm), could regulate live cell function nanoscopically. The genetically-encoded protein photosensitizers (engineered fluorescent proteins such as KillerRed, TagRFP, and flavin-binding proteins such as miniSOG, Pp2FbFP^L30M^) could be expressed in a cell type- and/or subcellular organelle-specific manner for targeted protein photo-oxidative activation/desensitization. The newly emerged active illumination technique provides an additional level of specificity. Typical examples of photodynamic *activation* include permanent activation of G protein-coupled receptor CCK1 and photodynamic activation of ionic channel TRPA1. Protein photosensitizers have been used to photodynamically modulate major cellular functions (such as neurotransmitter release and gene transcription) and animal behavior. Protein photosensitizers are increasingly used in photon-driven nanomanipulation in cell physiology research.

## Introduction

Photodynamic action as a physiological curiosity has a long history, dating back to more than a century ago (for an early review on this topic, please see Blum, 1932). Investigation of photodynamic modulation of cellular physiology, however, has been rather limited until the recent past. A number of technological advances in photodynamic research have been made in the past few years. The newly renovated photodynamic modulation is now poised to be used on a much wider scale in physiological research.

A typical photodynamic action involves light, light-absorbing organic molecule (photosensitizer, S), and oxygen. Singlet oxygen is generated in a Type II photodynamic action. The photosensitizer (S) after absorption of a photon (hυ) of appropriate wavelength is excited from ground state (S) to the singlet excited state (^1^S). The ^1^S then undergoes a physical process named intersystem crossing (isc), to reach the triplet excited state (^3^S). If the triplet state is sufficiently long-lived, its excitation energy can be transferred to the ground state molecular oxygen (^1^$\sum_{\text{g}}^{-}$), to generate the delta singlet oxygen (^1^Δ_g_)(^1^O_2_). The singlet oxygen so generated can react with cellular components (A), to trigger the full-scale cellular photodynamic responses (Cui and Matthews, 1998; Cui et al., 2012; Dai et al., 2012) (Scheme 1).

$$\begin{array}{l} \text{S}\overset{\text{hv}}{\to }{\;}^{1}\text{S}{\underset{k_{\text{isc}}}{\to }}^{3}\text{S}\overset{{\text{O}}_{2}}{\underset{k_{\text{O}}}{\to }}{\;}^{1}{\text{O}}_{2}\overset{\text{A}}{\underset{k_{\text{A}}}{\to }}{\text{AO}}_{2}\\ \;\\ \text{ SCHEME 1} \end{array}$$

Uncontrolled photodynamic action is detrimental as noted in porphyria patients (Kaestner et al., 2004; Norman, 2005), but measured photodynamic action has been utilized for major clinical advances. Concentrated generation of singlet oxygen at mega doses is cytocidal in different patterns (Agostinis et al., 2011; Krammer and Verwanger, 2012; Bacellar et al., 2015; Abrahamse and Hamblin, 2016). Singlet oxygen could trigger apoptosis, for example, by oxidizing multiple proteins (such as Bcl-2, Bcl-XL, BAX, BID) in the apoptosis pathway (Oleinick and Evans, 1998; Xue et al., 2001; Usuda et al., 2002, 2003; Chiu et al., 2005; Wan et al., 2008; Liu et al., 2011). Due to such cytocidal effects of singlet oxygen, photodynamic action has been found to be effective in the clinical treatments of both cancers and non-malignant lesions (Kennedy et al., 1990; Bown et al., 2002; Mittra and Singerman, 2002; Brown et al., 2004; Szeimies et al., 2010; Agostinis et al., 2011; Bown, 2013; Huggett et al., 2014; Craig et al., 2015; Abrahamse and Hamblin, 2016; Liu et al., 2016; Newman, 2016). On the other hand, it has been found that controlled doses of singlet oxygen could modulate cellular signaling in different cell types such as glandular cells with proven high specificity (Matthews and Cui, 1989, 1990a,b; al-Laith et al., 1993; Cui and Kanno, 1997; Cui et al., 1997, 2000, 2003, 2012; Cui and Matthews, 1998; Hashikura et al., 2001; Cui and Guo, 2002a,b; An et al., 2003; Wang et al., 2003; Krammer and Verwanger, 2012; Bacellar et al., 2015). One particular noted case is the photodynamic activation of CCK1 receptors in rat pancreatic acinar cells.

## Singlet oxygen and its permanent activation of CCK1 receptor

Singlet oxygen generated in photodynamic action with the sulphonated aluminum phthalocyanine (SALPC), has been found to activate rather permanently the CCK1 receptor in rat pancreatic acinar cells (Cui and Kanno, 1997; An et al., 2003; Cui et al., 2012), but desensitizes the α1 adrenergic receptor in rat hepatocytes (Cui et al., 2000) and other G protein-coupled receptors (GPCR).

**Figure 1:**
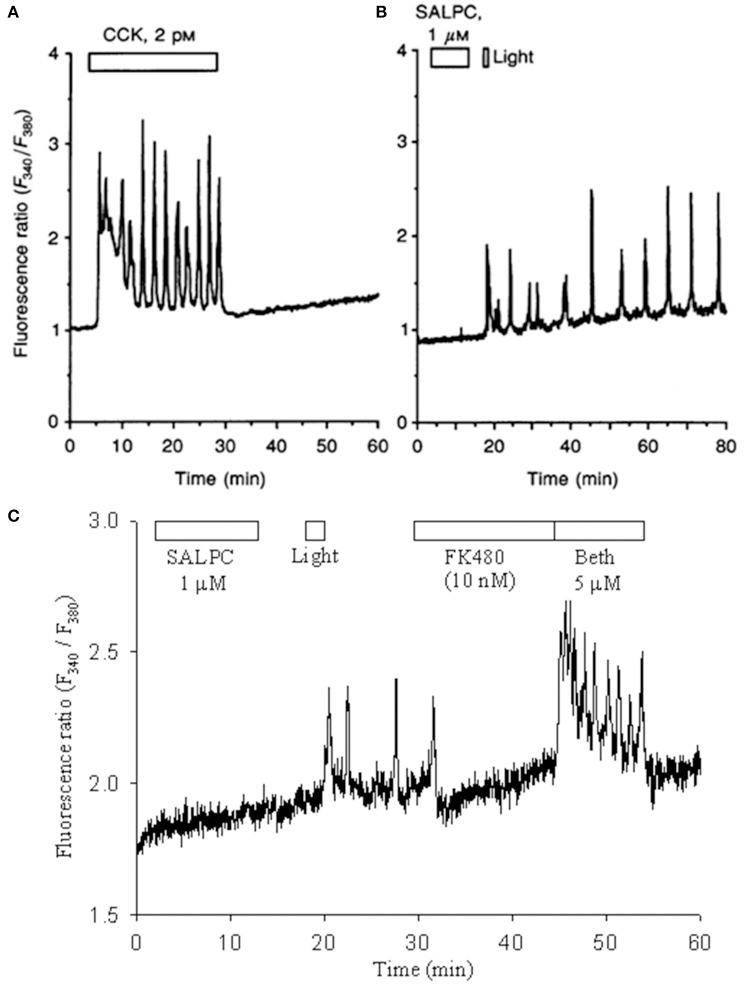
**SALPC photodynamic action triggers permanent activation of CCK1 receptors and persistent calcium oscillations in rat pancreratic acinar cells**. Fura-2 AM-loaded rat pancreatic acini were perifused, CCK **(A)**, sulphonated aluminium phthalocyanine (SALPC, **B,C**), CCK1R antagonist FK480 **(C)**, muscarinic agonist bethanechol (Beth, **C**), light illumination (λ > 580 nm) (55,000 lux, **B**; 53,000 lux or 72 mW/cm^2^, **C**) were applied, as indicated by the horizontal bars. Note that CCK-induced calcium oscillations ceased immediately after wash-out of CCK **(A)**, but photodynamically-induced calcium oscillations persisted even after photodynamic action **(B,C)**. The photodynamically-induced calcium oscillations were completely blocked by the CCK1R antagonist FK480, then stimulation with bethanechol still induced new regular calcium oscillations **(C)**. Reproduced from Cui and Kanno (1997) and An et al. (2003).

CCK-CCK receptors play important roles in both gastrointestinal (GI) and central nervous system (CNS) functions (Cawston and Miller, 2010; Yu and Smagghe, 2014), due to the wide-spread distribution of both CCK1 and CCK2 receptors (Miller and Gao, 2008; Dockray and Burdyga, 2011). Of the group A GPCR receptors, CCK1 is unique in that it can be permanently activated by photodynamic oxidation (Cui and Kanno, 1997; An et al., 2003; Cui et al., 2012). It may be noted that ligand (agonist CCK)-induced cytosolic calcium oscillations disappeared immediately after washout of CCK (Figure 1A), but the photodynamically-induced, ligand-independent calcium oscillations persisted well after photodynamic action (duration: 1 min) had completed (Figure 1B).

An early hint for irreversible activation of CCK1 receptor was noted as early as 1980 by the Jamieson group. A photoaffinity probe to label the CCK1 receptor, CCK octapeptide (*Asp-Tyr-(SO_3_H)-Met-Gly-Trp-Met-Asp-Phe-NH2, CCK-8*) analog 2-nitro-5-azidobenzoyl-Gly-*Asp-Tyr-(SO*_*3*_*H)-Met-Gly-Trp-Met-Asp-Phe-NH2* (NAB-Gly-*CCK-8*), was found to elicit irreversible secretion after UV irradiation of guinea pig pancreatic acini, although whether such secretion was mediated by the CCK1 receptor was not at that time verified (Galardy et al., 1980). A series of works by us have clearly delineated the irreversible nature of oxidative activation of CCK1 receptor (Matthews and Cui, 1989, 1990a,b; al-Laith et al., 1993; Cui et al., 1997, 2012; Cui and Kanno, 1997; An et al., 2003).

Photodynamic action with the photosensitizer sulphonated aluminum phthalocyanine (SALPC) was initially found to stimulate amylase secretion and regulate cytosolic signaling in the freshly isolated rat pancreatic acini (as reviewed in Cui and Matthews, 1998). In those experiments, the freshly isolated pancreatic acini were perifused, exposed to SALPC briefly (10 min), subsequent light illumination (2 min) then triggered persistent calcium oscillations which were completely blocked by CCK1 antagonist FK480 (10 nM) (An et al., 2003) (Figure 1C). After the blockade of calcium oscillations with FK480 (10 nM), the muscarinic agonist bethanechol (Beth) still triggered robust new calcium oscillations (An et al., 2003) (Figure 1C). These data indicated that after photodynamically-triggered calcium oscillations, pancreatic acinar cells *remained perfectly healthy* (An et al., 2003; Cui et al., 2012). Here the photodynamic action was restricted to the plasma membrane (Cui and Kanno, 1997; An et al., 2003; Cui et al., 2012).

## Protein photosensitizer for nanoscopically-confined photodynamic action

Singlet oxygen in the cellular *milieu* has a short lifetime (μs) (Cui and Matthews, 1998; Bovis et al., 2012; Kim et al., 2014), therefore has a limited effective diffusion distance of <10 nm (Moan and Berg, 1991; Cui and Matthews, 1998; Dougherty et al., 1998; Nowis et al., 2005; Cui et al., 2012). Singlet oxygen generated in photodynamic action is, therefore, effective only at the site of generation, or at the site of photosensitizer localization in the cell.

Due to their intrinsic physicochemical properties, photosensitizers of different chemical classes tend to accumulate preferentially at specific subcellular sites such as the plasma membrane, the endoplasmic reticulum (ER), lysosomes, mitochondria, to modulate cellular activities from their different subcellular locations after photodynamic action (Theodossiou et al., 2006; Allison and Sibata, 2010; Agostinis et al., 2011). Hematoporphyrin derivative (HPD) monomers tend to accumulate at the mitochondria, and HPD oligomers at the plasma membrane (Scourides et al., 1987). Mono-aspartyl chlorin e6 (MACE) after endocytosis localizes to lysosomes (Berg and Moan, 1997). Phthalocyanines tend to accumulate at mitochondria (Peng et al., 1991). Benzoporphyrin derivative (BpD) is localized to the Golgi apparatus (Rosenkranz et al., 2000). Protoporphyrin IX (PPIX) precursor ALA-synthesized PPIX distributes to the plasma membrane, lysosomes and mitochondria (Kennedy et al., 1990).

Interestingly, photodynamic action at different cellular sites triggers cell death by distinct pathways. Photodynamic action at mitochondria and lysosomes triggers apoptosis; photodynamic action at the ER elicits autophagy; whereas photodynamic action at the plasma membrane induces necrosis (Almeida et al., 2004; Bacellar et al., 2015; Abrahamse and Hamblin, 2016). As mentioned above, SALPC photodynamic action at the plasma membrane induced permanent activation of CCK1 receptor (Cui and Kanno, 1997; An et al., 2003; Cui et al., 2012), photodynamic action with Victoria Blue VO at mitochondria reduced the oscillatory frequency of receptor-mediated calcium oscillations (Cui and Guo, 2002a,b).

It is recognized that the specific localization of chemical photosensitizers are only relative, distribution in multiple subcellular sites are quite common (Kessel, 1997, 2004, 2012; Kessel et al., 1997; Oleinick and Evans, 1998). The plasma membrane-localized photosensitizer MCP, for example, is also found in the ER and lysosomes; photosensitizer SnOPA is present in the plasma membrane, but also in the ER, lysosomes and elsewhere (for an in-depth review, see Kessel, 2004).

Mutated fluorescent proteins or engineered flavin-binding proteins have significantly enhanced photosensitivity compared with their parental proteins. Such genetically-encoded protein photosensitizers can be targeted precisely to subcellular organelles after tagging with signal sequences or after fusion with target proteins, resulting in high-precision spatially-controlled photodynamic action, or targeted protein oxidation. Locally-expressed protein photosensitizers after absorption of photons at specified wavelength generate the highly reactive singlet oxygen. Singlet oxygen as noted above has limited diffusion distance (<10 nm), therefore modulates target proteins or subcellular organelles nanoscopically.

### Individual protein photosensitizers

**Figure 2:**
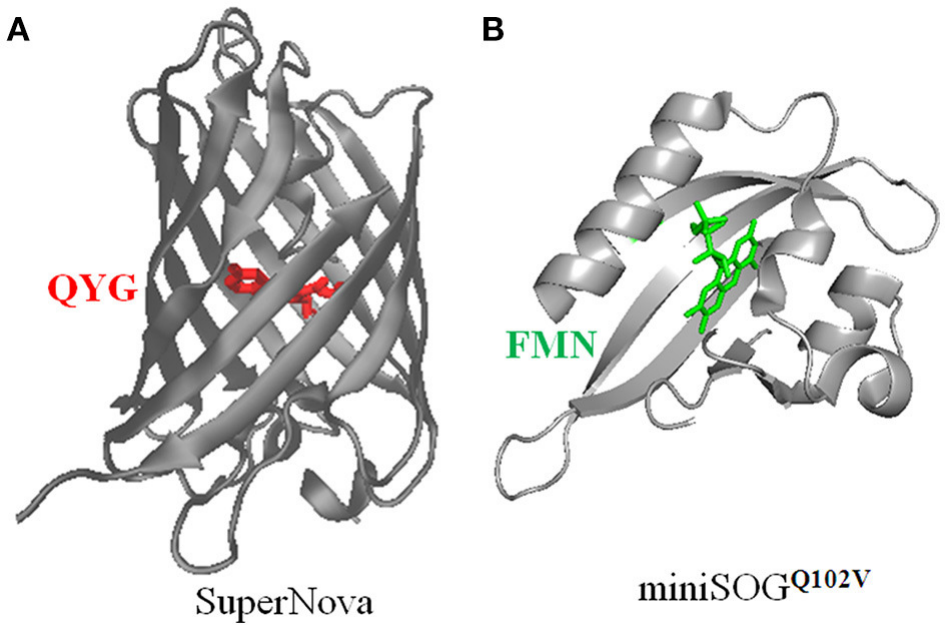
**Three dimensional structures of SuperNova (A)**, miniSOG^Q102V^ **(B)**, with their respective chromophores highlighted. **(A)** The full amino acid sequence of SuperNova is obtained from PDB database (3WCK). The three dimensional structure of SuperNova is from PDB in pdb format, input to VMD graphics. The chromophore of Gln65-Tyr66-Gly67 (Takemoto et al., 2013) in SuperNova is highlighted in red. **(B)** The full amino acid sequence of miniSOG^Q102V^ is from Rodríguez-Pulido et al. (2016). The sequence is put into the protein structure website Swiss-model, three-dimensional model is then obtained after a build-model step. The chromophore FMN in miniSOG^Q102V^ is highlighted in green. Model building similar to Mironova et al. (2013).

Protein photosensitizers are either fluorescent protein variants such as KillerRed, KillerOrange, TagRFP, or flavin-binding proteins such as miniSOG and variants miniSOG^Q102L/V^, and Pp2FbFP^L30M^. The miniSOG^Q102L^ is also named by some as singlet oxygen protein photosensitizer (SOPP). The chromophores (fluorophores) of KillerRed, KillerOrange, TagRFP are QYG, QWG, MYG, respectively, whereas miniSOG, miniSOG^Q102L/V^, Pp2FbFP^L30M^ all share the same chromophore of FMN (Figure 2).

#### KillerRed

The prototypical protein photosensitizer **KillerRed** is derived from the jellyfish chromoprotein anm2CP (239 residues, MWt 27 kD) (Bulina et al., 2006a,b; Pletnev et al., 2009), with point mutations of Thr145Asp, Cys161Gly (Shagin et al., 2004). KillerRed is composed of 11 anti-parallel β-sheets which form a barrel structure, with a central chromophore of Q65-Y66-G67 (see Figure 2) (Roy et al., 2010). An aqueous central channel/pore exists in the KillerRed structure, which is composed of the chromophore Q65-Y66-G67 and residues Ile142, Leu143, Pro144, Ile199, Ile200, Thr201. The excited chromophore can transfer its excitation energy to ground state molecular oxygen which has reached the chromophore region by diffusion through this channel; the generated ROS also exit KillerRed via the same channel (Carpentier et al., 2009; Pletnev et al., 2009; Serebrovskaya et al., 2009; Roy et al., 2010). The ROS quantum yield of KillerRed is more than 1000 times of EGFP (Bulina et al., 2006a,b; Carpentier et al., 2009; Pletnev et al., 2009). The current consensus is that KillerRed undergoes a Type I photodynamic action to generate superoxide anion, although it was previously thought to generate singlet oxygen by a Type II photodynamic action (Pletnev et al., 2009; Serebrovskaya et al., 2009; Shu et al., 2011; Vegh et al., 2011; Kim et al., 2014). A singlet oxygen-generating capacity cannot be completely ruled out, however (Roy et al., 2010; Petrova et al., 2016). Since KillerRed tends to dimerize, a monomeric mutant, **Supernova**, has been reported (Figure 2), which has the following 6 mutations compared with KillerRed: G3V, N145S, L160T, F162T, L172K, M204T (Takemoto et al., 2013). Supernova is believed to have similar photochemical properties as the parental KillerRed (Takemoto et al., 2013). The basic characteristics of KillerRed are listed in Table 1 (Bulina et al., 2006a,b; Lukyanov et al., 2010). Further mutations (G5C, Y68W, D119S, N147S, F179L, Y223H, E237Q) of KillerRed result in a blue-shifted **KillerOrange**, the photosensitization properties of KillerOrange remain to be investigated (Pletneva et al., 2015; Sarkisyan et al., 2015).

**Table 1:** **Basic properties of protein photosensitizers**.

**Photosensitizer**	**No. AA**	**λ_ex_ (nm)**	**λ_em_ (nm)**	**Φ^1^O_2_**	**Φ_fluo_**
KillerRed	239	585	610	(−)	0.25
KillerOrange	239	512	550	ND	0.42
TagRFP	237	555	584	0.004	0.48
miniSOG	106	448	500	0.03	0.45
Pp2FbFP^L30M^	148	449	495	0.09	0.25
miniSOG^Q102L/V^	106	440	487	0.25/0.39	0.43

*NO. AA, number of amino acid residues; λ_ex_, excitation wavelength; λ_em_, emission wavelength; Φ^1^O_2_, singlet oxygen quantum yield; Φ_fluo_, fluorescence quantum yield; (−), Only ${\text{O}}_{2}^{-.}$ is produced; ND, Not done*.

KillerRed fused with signal sequences can be targeted to the mitochondria in 293T cells (Bulina et al., 2006b), HEK293 cells, in the body wall muscle cells (Shibuya and Tsujimoto, 2012) and neurons of *C. elegans* (Williams et al., 2013). KillerRed can also be targeted to the lysosomes (Serebrovskaya et al., 2014), Golgi apparatus (Jarvela and Linstedt, 2014), the plasma membrane, mitochondria, or chromosomes (Shirmanova et al., 2013) in Hela cells. KillerRed has also been targeted to the plasma membrane in *C. elegans* neurons (Williams et al., 2013), zebrafish neurons and cardiomyocytes (Lee et al., 2010; Teh et al., 2010), or targeted to chromosomes in DU145 cells (Waldeck et al., 2011). The fusion protein TRF1-KillerRed has been used to target the telomeres (Sun et al., 2015). KillerRed fusion proteins (laminB1-KillerRed/histone2A-KillerRed) have been used to determine the spatial localization of chromosomal genes in the cell nucleus (Waldeck et al., 2013). The histone fusion protein H2B-KillerRed can be used to exert photodynamic blockade of cell division (Serebrovskaya et al., 2011; Shirmanova et al., 2015). Light irradiation of mitochondria-localized KillerRed (Mito-KillerRed) has been used to prune neuronal dendritic spines in defined dendritic regions of cultured neurons via the induction of caspase-3 activity (Ertuerk et al., 2014). Work done with larval zebrafish expressing KillerRed in the *habenula* afferent neurons from the ventral-lateral forebrain has helped to confirm that the habenula region is important for avoidance learning and helpless behavior (Lee et al., 2010).

#### TagRFP

**TagRFP** is derived from the *Entacmaea quadricolor* fluorescent protein TurboRFP (a random mutant of eqFP578), with mutations of R162E, Q166D, S180N, F198V, F200Y at the hydrophilic interface (Merzlyak et al., 2007). TagRFP has a central chromophore of M63-Y64-G65 (Subach et al., 2010). Light irradiated-TagRFP generates only ^1^O_2_, with a quantum yield of 0.004, but does not produce superoxide anion (Ragas et al., 2011). TagRFP has a high fluorescent quantum yield (Φ_fluo_ 0.48) and is widely used for fluorescent imaging (Merzlyak et al., 2007; Khrenova et al., 2015; Manoharan et al., 2015) (see Table 1). But when using TagRFP as a fluorescent probe for imaging, care must be taken that no undue photodamage is induced in either live cells or fixed tissue sections. Light illumination (532 nm, 40 mWatt/cm^2^) of TagRFP-expressing *E. coli* has been found to result in bacterial cell death (Ruiz-González et al., 2012).

#### miniSOG

The flavin-binding protein **miniSOG** is derived from the LOV2 domain of *A. thaliana* phototropin 2 with a Cys426Gly mutation, with further mutations S24G, I387M, N390S, S394T, F470L surrounding the chromophore (FMN) binding site (Shu et al., 2011). The resultant miniSOG is composed of 2 α-helix, interspersed with 5 β-sheets, with the chromophore FMN located between the α-helix and β-sheets (Pietra, 2014) (see Figure 2). Point mutation Cys426Gly facilitates transfer of FMN excitation energy absorbed from a photon to ground state O_2_ instead of to covalent bonding with Cys, significantly enhancing its ^1^O_2_ quantum yield (Shu et al., 2011). Initial report with anthracene-9, 10-dipropionic acid (ADPA) as the ^1^O_2_ probe obtained a quantum yield of 0.476 (Shu et al., 2011). Subsequent work has found that ADPA is also oxidized by other ROS (Ruiz-González et al., 2012). Direct measurement of ^1^O_2_ phosphorescence at 1275 nm, and the use of uric acid or PNS as ^1^O_2_ probes revealed a much lower quantum yield of 0.03 (Ruiz-González et al., 2012; Pimenta et al., 2013). The basic spectroscopic characteristics of miniSOG are listed in Table 1 (Shu et al., 2011; Ryumina et al., 2013; Wingen et al., 2014; Westberg et al., 2015).

Photosensitizer miniSOG can be targeted to the plasma membrane, mitochondria or chromosomes in Hela cells (as fusion proteins miniSOG-mem, miniSOG-mito, H2B-miniSOG) (Ryumina et al., 2013). In *C. elegans* motor neurons, miniSOG-TOMM-20 is targeted to mitochondrial outer membrane, whereas miniSOG-COX8a is targeted to mitochondrial matrix (Qi et al., 2012). miniSOG can be expressed as a fusion protein with the SDHC subunit (*mev-1*) of the mitochondrial respiratory chain complex II (succinate:ubiquinone oxidoreductase), to photodynamically inactivate with high specificity the respiratory chain complex II (on mitochondrial inner membrane) in *C. elegans*, without any damage toward its immediate neighbors complex I (NADH:ubiquinone oxidoreductase) or complex IV (cytochrome C oxidase) (Wojtovich et al., 2016). Photosensitizer miniSOG has also been fusion-expressed with vesicular SNARE proteins vesicule-associated membrane protein 2 (VAMP2) or synaptophysin 1 (SYP1), to target the small synaptic vesicles in cultured rat hippocampal neurons (Lin et al., 2013) or *C. elegans* neurons (Lin et al., 2013). miniSOG could be fusion-expressed with the synaptic active zone protein Munc13, to the pre-synaptic active zone in *C. elegans* neurons (Zhou et al., 2013).

#### Pp2FbFP^L30M^

**Pp2FbFP**^L30M^ is derived from the LOV domain of the flavin-binding protein Pp2FbFP from *Pseudomonas sputita*, with a further mutation of L30M (Torra et al., 2015). Measurement of phosphorescence at 1275 nm found that the ^1^O_2_ quantum yield of Pp2FbFP^L30M^ was 0.09 (Torra et al., 2015). The spectroscopic characteristics of Pp2FbFP^L30M^) are listed in Table 1 (Ryumina et al., 2013; Wingen et al., 2014).

#### SOPP

**miniSOG**^Q102L^ is also named SOPP, as mentioned above. In comparison with the parental miniSOG, in SOPP the FMN-binding glutamine is mutated to leucine (Q102L). This mutation reduces the hydrogen bond between Q102 and FMN, diminishing electron transfer, but enhancing energy transfer, with the net result of a much enhanced ^1^O_2_ quantum yield of 0.25 (Westberg et al., 2015, 2016). Another mutant, **miniSOG**^Q102V^ (Figure 2), has an even higher ^1^O_2_ quantum yield of 0.39 (Rodríguez-Pulido et al., 2016). When miniSOG^Q102L^ is expressed at the plasma membrane (by fusion with a PH domain) in *C. elegans* epithelial cells or in the cholinergic neurons, blue light illumination induces worm paralysis and neuronal injury, at efficiency higher than with miniSOG as the photosensitizer (Xu and Chisholm, 2016). The basic properties of miniSOG^Q102L^ are listed in Table 1. A new version of miniSOG, miniSOG2, involves seven point mutations: G22S, G40P, Q44R, R57H, L84F, H85R, M89I. Mutations R57H, Q44R, G40P, L84F directly interacting with the chromophore FMN are likely responsible for the red-shifted excitation and emission spectra, together with enhanced singlet oxygen generation, but the quantum yield remains to be measured (Makhijani et al., 2017).

### Targeted subcellular expression of protein photosensitizers

Subcellular targeting of protein photosensitizers has been done as mentioned above in different cell types, such as targeted KillerRed expression in Hela cells (Shirmanova et al., 2013; Jarvela and Linstedt, 2014; Serebrovskaya et al., 2014), HEK293 cells (Bulina et al., 2006b), DU145 cells (Waldeck et al., 2011), and miniSOG expression in Hela cells (Ryumina et al., 2013) or in cultured hippocampal neurons (Lin et al., 2013).

To target-express a protein photosensitizer, the photosensitizer gene needs to be fused with a subcellular localization sequence (SLS), to localize the protein photosensitizer to the desired subcellular compartments. KillerRed, for example, could be targeted to the plasma membrane (Bulina et al., 2006b; Teh et al., 2010) (plasma membrane LS, PMLS) with the N-terminal (20 residues) sequence of neuromodulin (Skene and Virág, 1989), or with the PH Delta1 sequence (Fujii et al., 1999; Bulina et al., 2006b). KillerRed can be targeted to mitochondria with the double sequences of MTS1 and MTS2 (Yang and Yang, 2011; Shibuya and Tsujimoto, 2012). Mitochondrial targeting sequence (MTS) can be derived from human cytochrome *C* oxygenase VIII subunit (Rizzuto et al., 1989, 1995), or from members of the respiratory chain complexes (Wojtovich et al., 2016). For lysosomal targeting, the C-terminal cytosolic tail sequence of the lysosomal-associated membrane protein II (LIMP II) could be used (Tabuchi et al., 2000). GTPase Rab7A sequence has been used to target KillerRed to lysosomes (Serebrovskaya et al., 2011; Ryumina et al., 2016). ER targeting could use the ER localization sequence (MLLSVPLLLGLLGLAVA) of calreticulin (Fliegel et al., 1989) and the ER retaining sequence KDEL (Munro and Pelham, 1987). The human β-1,4-galactotransferase N-terminal sequence can be used to target TagRFP to the Golgi apparatus (Shaner et al., 2008). Fusion proteins miniSOG-H2B, miniSOG-VAMP2, SYP1-miniSOG as mentioned above target miniSOG to Hela cell chromosomes (Ryumina et al., 2013), and to the small synaptic vesicles, respectively (Lin et al., 2013). KillerRed-TRF1 targets to the telomeres (Sun et al., 2015). The complete amino acid sequence of protein photosensitizers are listed in Table 2.

**Table 2:** **Amino acid sequence of protein photosensitizers**.

**KillerRed (Bulina et al., 2006a)**						
1	MGSEGGPALF	QSDMTFKIFI	DGEVNGQKFT	IVADGSSKFP	HGDFNVHAVC	ETGKLPMSWK
61	PICHLI**QYG**E	PFFARYPDGI	SHFAQECFPE	GLSIDRTVRF	ENDGTMTSHH	TYELDDTCVV
121	SRITVNCDGF	QPDGPIMRDQ	LVDILPNETH	MFPHGPNAVR	QLAFIGFTTA	DGGLMMGHFD
181	SKMTFNGSRA	IEIPGPHFVT	IITKQMRDTS	DKRDHVCQRE	VAYAHSVPRI	TSAIGSDED
**SuperNova (Takemoto et al., 2013)**						
1	MGSE**V**GPALF	QSDMTFKIFI	DGEVNGQKFT	IVADGSSKFP	HGDFNVHAVC	ETGKLPMSWK
61	PICHLI**QYG**E	PFFARYPDGI	SHFAQECFPE	GLSIDRTVRF	ENDGTMTSHH	TYELDDTCVV
121	SRITVNCDGF	QPDGPIMRDQ	LVDILP**S**ETH	MFPHGPNAVR	Q**T**A**T**IGFTTA	DGG**K**MMGHFD
181	SKMTFNGSRA	IEIPGPHFVT	IITKQ**T**RDTS	DKRDHVCQRE	VAYAHSVPRI	TSAIGSDED
**KillerOrange (Pletneva et al., 2015)**						
1	MGSE**C**GPALF	QSDMTFKIFI	DGEVNGQKFT	IVADGSSKFP	HGDFNVHAVC	ETGKLPMSWK
61	PICHLI**QWG**E	PFFARYPDGI	SHFAQECFPE	GLSIDRTVRF	ENDGTMTSHH	TYEL**S**DTCVV
121	SRITVNCDGF	QPDGPIMRDQ	LVDILP**S**ETH	MFPHGPNAVR	QLAFIGFTTA	DGGLMMGH**L**D
181	SKMTFNGSRA	IEIPGPHFVT	IITKQMRDTS	DKRDHVCQRE	VA**H**AHSVPRI	TSAIGSD**Q**D
**TagRFP (Ruiz-González et al., 2012)**						
1	MVSKGEELIK	ENMHMKLYME	GTVNNHHFKC	TSEGEGKPYE	GTQTMRIKVV	EGGPLPFAFD
61	ILATSF**MYG**S	RTFINHTQGI	PDFFKQSFPE	GFTWERVTTY	EDGGVLTATQ	DTSLQDGCLI
121	YNVKIRGVNF	PSNGPVMQKK	TLGWEANTEM	LYPADGGLEG	RSDMALKLVG	GGHLICNFKT
181	TYRSKKPAKN	LKMPGVYYVD	HRLERIKEAD	KETYVEQHEV	AVARYCDLPS	KLGHKLN
**miniSOG (Shu et al., 2011)**						
1	MEKSFVITDP	RLPDNPIIFA	SDGFLELTEY	SREEILGRNG	RFLQGPETDQ	ATVQKIRDAI
61	RDQREITVQL	INYTKSGKKF	WNLLHLQPMR	DQKGELQYFI	GV**Q**LDG	
**Pp2FbFP^L30M^ (Torra et al., 2015)**						
1	MINAKLLQLM	VEHANDGIVV	AEQEGNESI**M**	IYVNPAFERL	TGYCADDILY	QDARFLCGED
61	HDQDGIAIIR	EAIREGRPCC	QVLRNYRKDG	SLFWNELSIT	PVHNEADQLT	YYIGIQRDVT
121	AQVFAEERVR	ELEAEVAELR	RQQGQAKH			
**SOPP/miniSOG^Q102L/V^ (Westberg et al., 2015; Rodríguez-Pulido et al., 2016)**						
1	MEKSFVITDP	RLPDNPIIFA	SDGFLELTEY	SREEILGRNG	RFLQGPETDQ	ATVQKIRDAI
61	RDQREITVQL	INYTKSGKKF	WNLLHLQPMR	DQKGELQYFI	GV**L**LDG	

*Protein photosensitizers (noted residues are shown in bold type and underlined, for details see text)*.

For whole organism studies, it is routine to place the desired gene under cell type- or tissue-specific promoters. Place the protein photosensitizer gene or gene construct under a suitable promoter, the sensitizer could then be expressed in that tissue (neuron, muscle, for example) only, in model animals *C. elegans*, zebrafish or others at the desired subcellular compartments (Serebrovskaya et al., 2011, 2014; Kobayashi et al., 2013; Williams et al., 2013). Cell-type specific viral vectors are also useful for *in vivo* injections. Engineered Muller cell-specific adenovirus variant containing the KillerRed gene, ShH10-KillerRed, for example, has been used to target the mouse retinal Muller cells. This study has confirmed the essential roles of Muller cells in visual perception and in normal retinal structure formation (Byrne et al., 2013). In this regard some technical strategies for gene delivery (Kaestner et al., 2015a; El-Shamayleh et al., 2016) and past works on tissue-specific expression of fluorescent protein sensors may be considered (Akemann et al., 2013; Kaestner et al., 2015b).

### Selective illumination of defined regions for localized photodynamic action

Other than subcellularlly-defined expression of protein photosensitizers, photodynamic action could be further spatially-delimited with ultra-structurally distinct point illumination. The recently emerged active illumination (AI) technology can be done at single or dual wavelengths, in multiple cells or cellular regions, simultaneously with imaging. The illumination light spot could vary in size, shape, and light intensity, with the spot size down to the theoretical diffraction limit (0.2 × 0.2 μm) (Shkryl et al., 2012). Active or selective illumination is made possible due to the invention of digital micromirror arrays, which can in real-time control the angle of each micromirror in the array (Shkryl et al., 2012).

Such selective illumination has been used to un-cage calcium or inositol 1, 4, 5-triphosphate (IP_3_) at multiple cellular sites simultaneously (Shkryl et al., 2012). Selective dual-wavelength illumination (390, 510 nm) could open or close, in tandem, designer photosensitive potassium channels in neurons (Janovjak et al., 2010). It has been shown that after selective irradiation of cultured vascular endothelial cells, localized photodynamic action readily triggered focused cell death in the irradiated areas (Feine et al., 2012). In channelrhodopsin 2 (ChR2)-expressing *C. elegans*, selective illumination of dendrites of sensory neurons stimulated cytosolic calcium increase, leading to enhanced worm activities (Cho and Sternberg, 2014).

## Highlighted examples of photodynamic modulation of cellular physiology

Photodynamic modulation of cellular functions with protein photosensitizers is outlined above. Works in the following areas have emerged which are of particular significance in cellular physiology.

### Modulation of ionic channels

The earliest example of photodynamic modulation of native ionic channels is illustrated by the photodynamic blockade, with photosensitizers Rose Bengal and Eosin Y, of voltage-gated sodium channels (Na_v_) in the squid giant axons. In addition, photodynamic action also slowed or disrupted Na_v_ inactivation (Oxford et al., 1977). Photodynamic action with Rose Bengal has also been found to inhibit other voltage-gated channels as well (Na_v_, K_v_, Ca_v_) in the isolated frog (*Rana pipiens*) atrial cardiomyocyte. After photodynamic action, Na_v_ inactivation was significantly slowed; Ca_v_ inactivation was also inhibited (Tarr and Valenzeno, 1991). Photodynamic action with Rose Bengal was found to inhibit Ca_v_, K_v_, K_Ca_ channels in rat anterior pituitary cells GH3 (Valenzeno and Tarr, 1997).

K_v_ channels expressed in cell lines have been found to be inhibited by photodynamic action with porphyrins (photosensitizer) conjugated to subtype-specific monoclonal antibodies. Photodynamic action with anti-Kv4.2 mAb-porphyrin conjugates was found to facilitate photoablation of Kv4.2, but not of Kv4.3 or Kv2.1 (Sack et al., 2013). Similarly photodynamic action (laser light at 473 nm, 350 mW/cm^2^) with channel-binding photosensitizer FITC-cAMP (bound to CNBD in mHCN2) was found to inhibit mouse potassium channel mHCN2 expressed in *Xenopus laevis* oocytes. Photodynamic modulation of mHCN2 in the closed state decreased subsequently induced I_h_ current significantly. In contrast, photodynamic action enhanced the I_inst_ current and delayed channel inactivation when photosensitization was executed in the open channels (Gao et al., 2014). These latter two photodynamically-induced changes were determined to be due to oxidation or *cross-linking* of the His434 residue located at the cytosolic side of the S6 segment, because H434A mutation abolished the delay in channel deactivation, and the generation of I_inst_ (Gao et al., 2014). Interestingly, when miniSOG was fused to the C terminal end (C terminal end of CNBD) of mHCN2, light irradiation was found to exert (on mHCN2) effects similar to those observed with the chemical photosensitizer FITC-cAMP (Gao et al., 2014).

**Figure 3:**
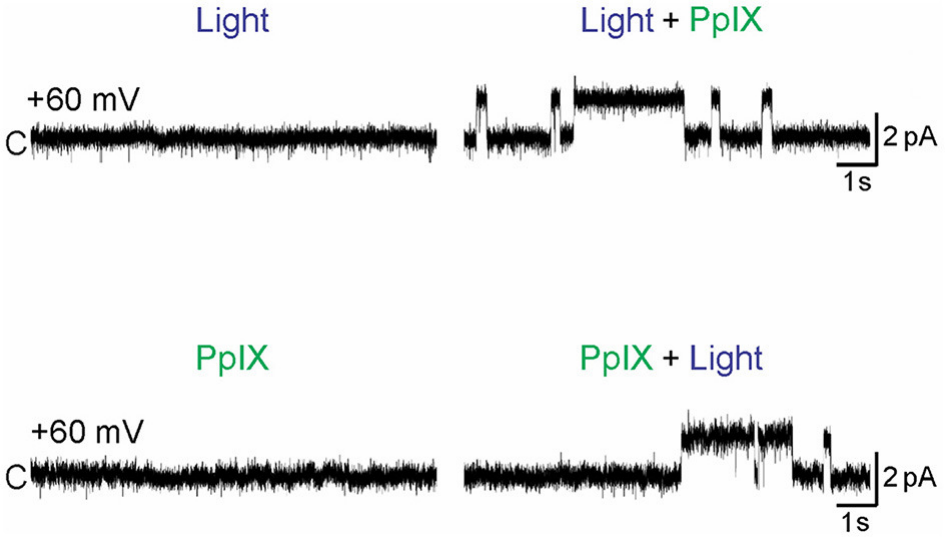
**Photodynamic activation of TRPA1**. Purified human TRPA1 reconstituted into artificial lipid (1,2-diphytanoyl-snglycero-3-phosphocholine: cholesterol was 9:1) bilayers with both the N- and C-terminals facing the cytosol were voltage-clamped at + 60 mV. PPIX (1 μM) was added to the cytosolic side, laser light (405 nm) was applied (at 0.45 mW/mm^2^). Single channel currents were measured using Patch-a-Patch in symmetrical K^+^ solution (150 mM KCl, 10 mM HEPES) under light illumination alone (Light), protoporphyrin IX alone (PPIX), or PPIX plus light (Light + PPIX) as indicated. Scale bars indicate 2 pA current and 1 s. Only simultaneous presence of light and PPIX (photodynamic action) led to channel opening. From Babes et al. (2016).

In contrast to the inhibitory effect on the voltage-gated channels Na_v_ and K_v_, photo-oxidation has been found to *activate* the calcium-permeant sensory channels TRPA1 and TRPV1 (Hill and Schaefer, 2009; Babes et al., 2016). Photodynamic action with photosensitizers acridine orange (490 nm), or hypericin (590 nm) was found to activate TRPA1 expressed in HEK293 cells (Hill and Schaefer, 2009). Photodynamic action with photosensitizer protoporphyrin IX was found to drastically activate both TRPA1 and TRPV1. Purified human TRPA1 inserted in artificial lipid bilayers was found to be activated only after photodynamic action with protoporphyrin IX and blue light (Babes et al., 2016) (Figure 3). Similar works involving the protein photosensitizers both *ex vivo* and *in vivo* are eagerly awaited.

### Modulation of the exocytotic SNARE complex

**Figure 4:**
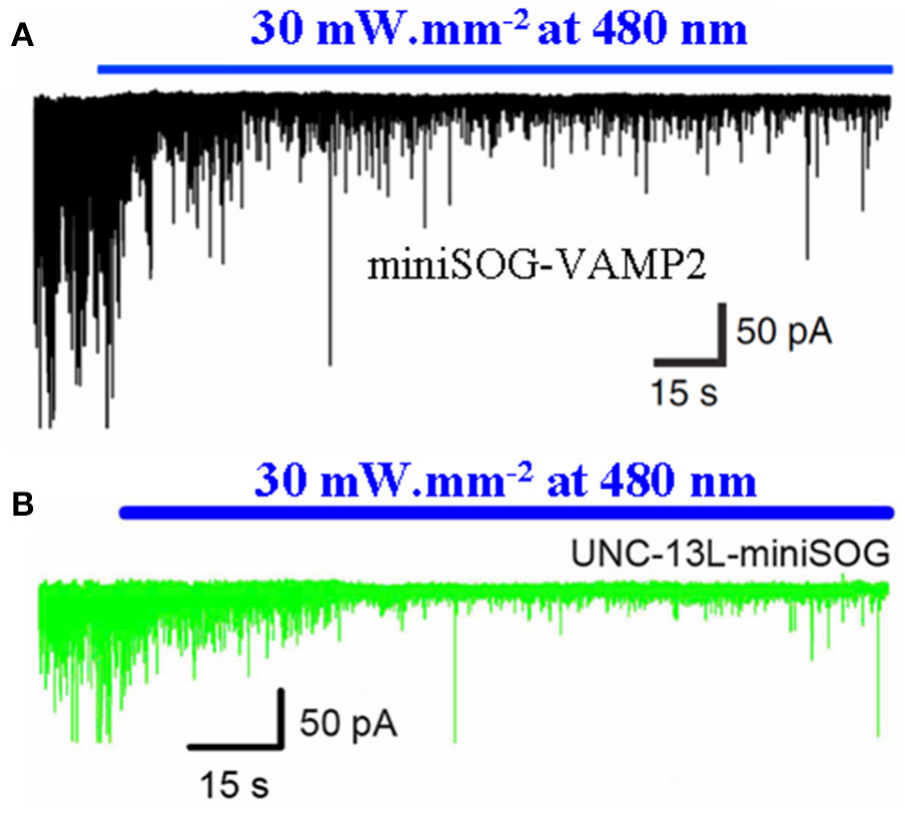
**Photodynamic inhibition of neurotransmitter release in ***C. elegans*** with miniSOG-VAMP2 or UNC-13L-miniSOG**. The protein photosensitizer miniSOG was fusion-expressed with the vSNARE vesicular associated membrane protein 2 (VAMP2) **(A)** or with the active zone protein UNC-13-L (Munc 13) **(B)** in *C. elegans* neurons under neuron-specific promoter. The transgenic worms were illuminated with blue light (480 nm, 30 mW/mm^2^), and postsynaptic current was recorded from ventral medial wall body muscle with whole cell configuration. Note the rapid decrease in excitatory postsynaptic current upon blue light illumination. Adapted from Lin et al. (2013) and Zhou et al. (2013).

The SNARE complex proteins and associated synaptic active zone proteins are essential for regulated neurotransmitter release. Protein photosensitizer miniSOG fused with the v-SNARE proteins VAMP2 or synaptophysin (miniSOG-VAMP2, SYP1-miniSOG) was expressed in neonatal rat hippocampal slices or in cultured hippocampal and cortical neurons. Transgenic *C. elegans* worms with panneuronal expression of miniSOG-VAMP2 were also made. The fused miniSOG (miniSOG-VAMP2, SYP1-miniSOG) was found to localize to the synaptic vesicles, but without any effect on transmitter release or on animal behavior in the dark. Upon illumination with blue light (480 nm), neurotransmitter release in cultured neurons or in hippocampal slices were completely blocked, with the inhibition lasting for >1 h (Lin et al., 2013). In *C. elegans* panneuronally expressing miniSOG-VAMP2, light irradiation similarly led to marked inhibition of spontaneous neurotransmitter release, with reduced movement and worm paralysis (Lin et al., 2013). Light irradiation (480 nm) of *C. elegans* expressing miniSOG fused to the pre-synaptic active zone protein Munc13 led to similar acute inhibition of neurotransmitter release (Zhou et al., 2013) (Figure 4).

### Modulation of nuclear events

**Figure 5:**
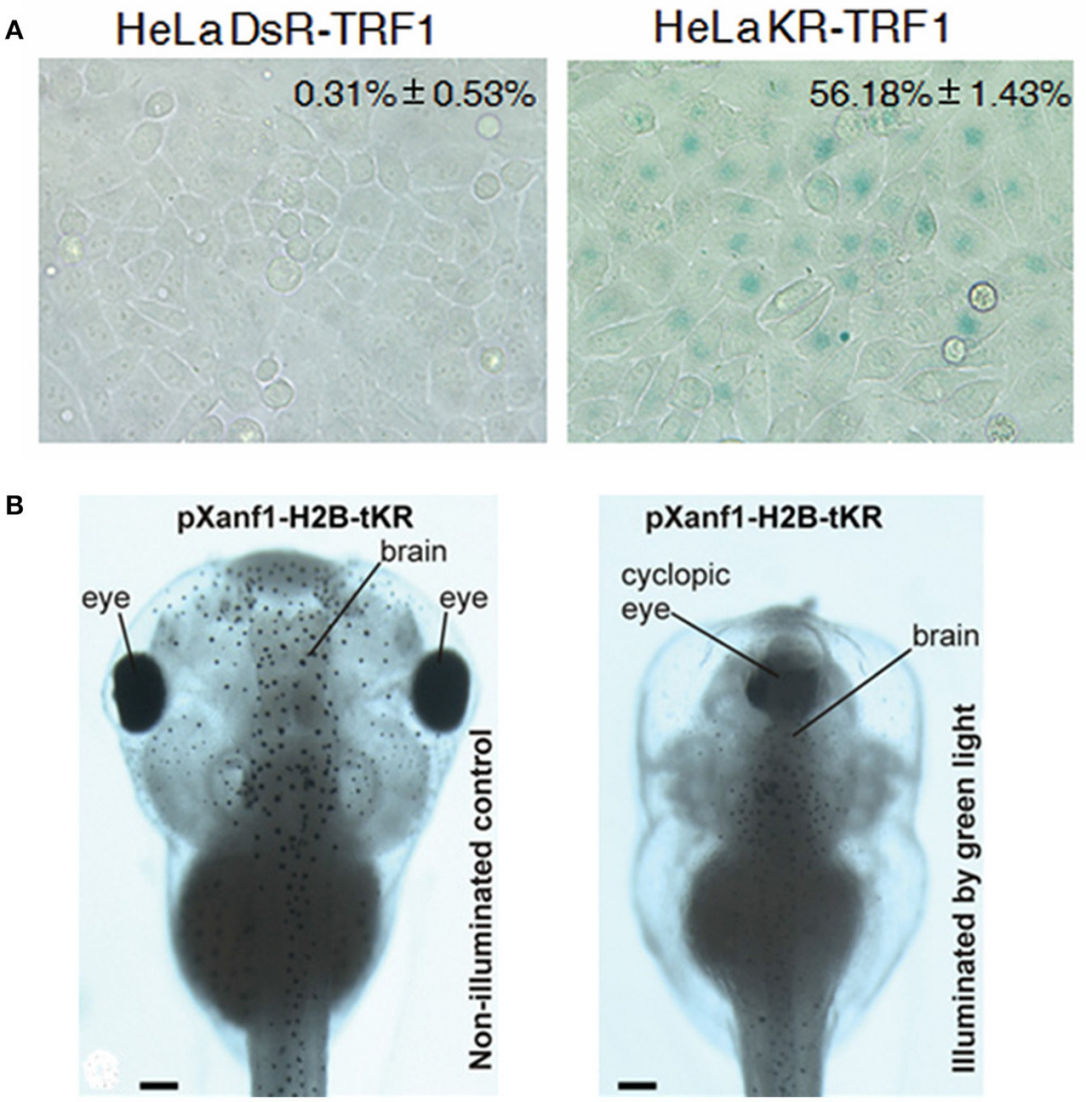
**Photodynamic transformation of cell fate and zebrafish development with KillerRed fusion-expressed with TRF1 at the telomere (KR-TRF1) in HeLa cells or with H2B under the promoter ***pXanf1*** in ***X. laevis***. (A)** KillerRed (KR) was fusion-expressed with telomere repeat binding factor 1 (TRF1) to Hela cell telomeres (with the non-photosensitizer DsRed used as control), repeated light exposure (1.5 mW/cm^2^, 10 min in each passage for 30 passages) induced fastened cell senescence as indicated by enhanced β–galactosidase activity (percentage cells with SA-β-gal staining). The negative control fluorescent protein DsR showed no such effect. **(B)** KillerRed was fusion-expressed with core histone H2B under the control of forebrain promoter *pXanf1*, the transgenic embryos were illuminated at the early midneurula stages with green LED (525 nm, 45 mW/cm^2^, 1 h). Such photodynamic treatment severely retarded the development of the forebrain, sometimes resulted in a completely cyclopic phenotype, similar to phenotypes with suppressed *pXanf1* expression (illuminated by green light). The tadpoles developed from transgenic *X. laevis* embryos not illuminated showed normal phenotype (non-illuminated control). Scale bars in **(B)** 100 μm. Adapted from Sun et al. (2015) and Serebrovskaya et al. (2011).

Another place of interest that has been investigated is the chromosomal tip, the telomeres. The photosensitizer KillerRed could be fusion-expressed with the telomere repeat binding factor 1 (KillerRed-TRF1). The spatially-defined generation of singlet oxygen after photodynamic action at the telomeres was found to result in telomere abnormalities (telomere associations, shortened or complete loss of telomeres), leading to fastened cell senescence or cell death in cultured cancer cells HeLa, U2OS and IMR90 (Sun et al., 2015) (Figure 5A). KillerRed fusion expressed with the chromosomal protein histone H2B (H2B-KR-KR) in adherent HeLa-Kyoto cells after green light illumination was found to induce acute wide spread damages to genomic DNA, leading to non-separation of chromosomes, and to blockade of cell division and proliferation. H2B-KR-KR target expressed in specific tissues (under control of tissue-specific promoters) in *Xenopus* embryos was found, after green light illumination (540–580 nm 120 mW/cm^2^; 525 nm, 45 mW/cm^2^), to retard organogenesis. Therefore, H2B-KR-KR photodynamic action could be used to study cell division, organism development, organogenesis or carcinogenesis in a cell-specific manner *in vivo* (Serebrovskaya et al., 2011) (Figure 5B). Protein photosensitizers tandem-KillerRed and miniSOG target-expressed at chromatin (H2B-tKR or H2B-miniSOG) in HeLa-Kyoto cells after brief light irradiation (H2B-tandem KillerRed expressing cells with green light 540–580 nm for 15 min at 200 mW/cm^2^; H2B-miniSOG for 5 min with blue light 465–495 nm at 65 mW/cm^2^) was able to induce single strand breaks but only miniSOG induced double strand breaks of the genomic DNA, leading to DNA damage response and cell senescence (Petrova et al., 2016). Most interestingly, germline C. elegans expressing Histone-mSOG after exposure to blue light has been found to produce progenies with inheritable phenotypes (Noma and Jin, 2015). KillerRed fusion-expressed either with the peripheral nuclear protein lamin B1 (KRed-Lamin B1) or with the diffusely distributed chromosomal protein H2A (H2A-KRed) was used to identify gene damages after photodynamic action. The extent of photo-oxidative damage was used to delineate the spatial localization of the respective genes and the gene-carrying chromosomes in the nucleus (Waldeck et al., 2013). KillerRed fused to a tet-repressor (tetR-KR) or a transcription activator (TA-KR) have been used to target oxidative stress to hetero- or euchromatin respectively in U2OS cells (Lan et al., 2014).

The above examples showcase targeted photonanomanipulation in subcellular organelle-based events at the plasma membrane, synaptic vesicles and at chromosomes. Numerous other subcellular sites can be similarly modulated with this light-controlled nanomanipulation technique.

## Conclusion and perspectives

In conclusion, the emergence of protein photosensitizers with enhanced singlet oxygen quantum yield has made possible the fast-track progress in photodynamic nanomanipulation of cellular physiology, mainly in *C. elegans* and zebrafish, whole organism works in mammalian animals remain to be expanded. The targeted expression after insertion of signaling sequence, fusion expression of target proteins, or promoter-driven tissue-specific expression, have made possible highly confined generation of singlet oxygen, with targeted nanoscopical protein oxidative activation or inactivation. Of the three major photopharmacological techniques available today—optogenetics involving channelrhodopsin and other photosensory domain-containing proteins spatial configurational-responsive to the absorption of a photon, photochromic ligands of receptors or ionic channels involving the photon-driven configurational changes of small molecule ligands, and photodynamic modulation involving photosensitizer-generated singlet oxygen, the protein photosensitizers are unique in that they could be repetitively irradiated (used) rather like an enzyme molecule to generate multiple copies of singlet oxygen molecules but not merely repetitive configuration changes in the light-absorbing molecules themselves. This makes possible multiple-hit oxidative activation or desensitization of receptors and channels important in cellular physiology, with the use of attenuated lasers and LED, or with cheap halogen cold light sources. In the future, more extensive works may be done with this photonanomanipulation technique both in the elucidation of basic cellular functions such as gene transcription, protein synthesis, transport, degradation, or in the elucidation of cell-type specific functions in the central nervous system circuits or in the innervation hubs (ganglia) and pathways (nerve fiber wirings) of the peripheral nervous system. Such photonanomanipulations *in situ* could also be used to study the intrinsic long-term repair mechanisms with high spatial and temporal resolutions.

## Author contributions

HJ and YL wrote the initial drafts. ZC conceived the idea of the review and finalized the last version of the article. All authors checked and approved the submitted version.

## Funding

Work in the authors' laboratory has been supported by grants from The Natural Science Foundation of China (Nos. 31670856, 31270892) and The National Basic Research Program (973 Program) from The Ministry of Science and Technology of China (No. 2011CB809101).

### Conflict of interest statement

The authors declare that the research was conducted in the absence of any commercial or financial relationships that could be construed as a potential conflict of interest.
